# Digital Twins Supporting Efficient Digital Industrial Transformation

**DOI:** 10.3390/s21206829

**Published:** 2021-10-14

**Authors:** Dinithi Bamunuarachchi, Dimitrios Georgakopoulos, Abhik Banerjee, Prem Prakash Jayaraman

**Affiliations:** Department of Computer Science and Software Engineering, Swinburne University of Technology, Hawthorn 3122, Australia; mbamunuarachchi@swin.edu.au (D.B.); dgeorgakopoulos@swin.edu.au (D.G.); abanerjee@swin.edu.au (A.B.)

**Keywords:** cyber twins, digital twins, Industry 4.0 cost model

## Abstract

Industry 4.0 applications help digital industrial transformation to be achieved through smart, data-driven solutions that improve production efficiency, product consistency, preventive maintenance, and the logistics of industrial applications and related supply chains. To enable and accelerate digital industrial transformation, it is vital to support cost-efficient Industry 4.0 application development. However, the development of such Industry 4.0 applications is currently expensive due to the limitations of existing IoT platforms in representing complex industrial machines, the support of only production line-based application testing, and the lack of cost models for application cost/benefit analysis. In this paper, we propose the use of Cyber Twins (CTs), an extension of Digital Twins, to support cost-efficient Industry 4.0 application development. CTs provide semantic descriptions of the machines they represent and incorporate machine simulators that enable application testing without any production line risk and cost. This paper focuses on CT-based Industry 4.0 application development and the related cost models. Via a case study of a CT-based Industry 4.0 application from the dairy industry, the paper shows that CT-based Industry 4.0 applications can be developed with approximately 60% of the cost of IoT platform-based application development.

## 1. Introduction

Industry 4.0 is the latest trend in advanced manufacturing, and it is powered by advancements in the Internet of Things (IoT) and Artificial Intelligence (AI) [1]. Industry 4.0 applications are helping the digital industrial transformation and use IoT-based solutions to integrate and interact with machines to harvest their data, AI-based solutions to analyze machine data, and the combination of these foundation technologies for the production of high-value information in the form of recommendations or decisions that improve manufacturing production efficiency, reduce unplanned maintenance, and enhance product consistency [2,3].

Industry 4.0 applications utilize complex machines and their development using conventional IoT platforms is costly and time-consuming. This is because, unlike IoT devices that are relatively simple machines that often incorporate a single sensor or actuator, a complex industrial machine includes considerable built-in automation and incorporates many sensors and actuators. Therefore, integrating and interacting with complex machines involves considerable cost and time to (1) analyze such machines to identify the machine data parameters that need to be integrated with each Industry 4.0 application, and (2) integrate the machines with an existing IoT platform (e.g., Azure IoT) that will be used to develop the Industry 4.0 applications. Furthermore, machine data integration may also require the translation of machine data into meaningful information related to the context of the application which demands additional work. Finally, testing the Industry 4.0 applications that utilize complex machines is both challenging and extremely time-consuming and costly, as it requires the use of the actual machines and entails a risk of potential unplanned stoppages and machine damage. The integration and use of machine simulators for testing purposes are extremely important for Industry 4.0 application testing. In addition, updating and porting an existing application to a machine change or across a manufacturing plant requires a considerable amount of rework.

To explain these, let us consider an example from the food manufacturing industry. The production process of food spreads (such as jam, vegemite, tomato paste, peanut butter) involves the utilization of evaporator machines to process the raw materials to achieve the targeted quality and the consistency of the final product [4]. To achieve the target product quality, machine operators often need to adjust the machine settings that control the product consistency (e.g., the steam pressure, inflow, outflow, and other parameters) of the evaporator machines. Industry 4.0 applications can automate and increase product consistency by determining and recommending the optimum machine settings that will achieve or exceed the product quality targets set by the manufacturer by (1) analyzing the machine sensor data (e.g., pressure, temperature) as well as the current machine settings (e.g., the setting/position of its control valves), and (2) computing the optimal machine settings that will achieve the target product quality and applying these to the evaporator machine. Furthermore, Industry 4.0 application testing often involves using evaporator machine simulators to test the relevant aspects of the machine’s automation before the application is deployed in the plant to avoid machine damage and to reduce plant downtime. Any upgrade of the evaporator machine utilized by an existing Industry 4.0 application or porting an existing application to a different plant further requires the above integration and testing activities to be performed for the new machines.

Currently, developing such an Industry 4.0 application using existing industrial IoT platforms is expensive and time-consuming. The lack of standardized and semantically rich machine descriptions impedes efficient application development using complex machines [5,6] and identifying the machine data needed by an application generally requires going through the machine manuals. Moreover, this needs to be repeated each time when a new Industry 4.0 application needs to be introduced into the plant. Machine data integration with the application requires first integrating the machines with an IoT platform that would be used to develop the application. However, if the integration is application-specific, it may have a limited ability to support a new application that needs to use the same machine [7]. Moreover, existing support for the reuse of the machine data translation functionalities is limited and results in considerable rework to support similar applications [8]. Furthermore, Industry 4.0 application testing using the existing IoT platforms requires knowledge about the behaviors of the complex machines including machine automation for enabling device simulation [9]. Thus, application testing consumes a considerable amount of time. In addition, updating an application to a change of a machine or porting an application to a different plant is extremely costly due to the required rework, even when the new plant has similar types of machines.

Digital Twins (DTs) are digital representations of physical objects (processes or systems). DTs and their corresponding physical objects have a bi-directional data flow between them, and DTs closely reflect the state and the behavior of the corresponding physical object [10,11,12]. In the context of Industry 4.0, DTs are increasingly being used to represent industrial machines and to provide smart data-driven solutions in manufacturing plants [13], including for predictive maintenance, product quality monitoring [14,15], and production process optimizations [16,17,18,19,20]. However, there are limitations in existing DTs and their ability to support cost-efficient Industry 4.0 application development, testing, update, and porting. A detailed analysis of the related work, including that of the DTs, is presented in Section 2. The proposed Cyber Twins (CTs) [7], are an extension of existing DTs and they address some of the limitations in existing DTs to support cost-efficient Industry 4.0 application development, testing, update, and porting. CTs can be cost efficiently auto-generated by using the machine semantic descriptions and they contain semantic descriptions of the machines and the CTs themselves. In addition, a CT can be cost efficiently updated to accommodate machine upgrades or replacements, as well as provide services to support application testing by incorporating a simulator of the corresponding machine. In [21], we proposed a CT Management Framework for generating and managing CTs.

In this paper, we focus on CTs and their use in CT-based Industry 4.0 application development and propose a cost model that captures Industry 4.0 application development costs. Next, we use these to compare the CT-based Industry 4.0 application development with the current state-of-the-art IoT platform-based approach for the development of Industry 4.0 applications. Finally, we show that CT-based Industry 4.0 application development is more cost-efficient via a case study from the dairy industry. The main novel contributions of this paper are the following:

- The CTs, and in particular, the ability of each CT to digitally represent a specific complex industrial machine via a semantic description of the machine, to communicate with the corresponding machines to obtain its data and send machine settings and other actuation to the machine, and to simulate the machine operation to facilitate Industry 4.0 application testing;
- CT-based Industry 4.0 application development that utilizes CTs instead of individual sensors and actuators to make Industry 4.0 application development more cost-efficient. Unlike IoT platform-based Industry 4.0 application development that can integrate the individual sensors and actuators of the machine, CT-based application development can integrate entire-complex machines reducing the cost of application development, especially when CTs are used by multiple Industry 4.0 applications;
- A novel cost model for estimating the cost of developing an Industry 4.0 application. To the best of our knowledge, no other model currently exists for that.
- An evaluation of CT-based applications using a sample application from the dairy industry that shows the benefits of CT-based Industry 4.0 application development.

The rest of the paper is organized as follows. Section 2 presents the related work and Section 3 presents an overview of the CTs. Section 4 describes CT-based Industry 4.0 application development and presents the cost model proposed for modelling Industry 4.0 application development costs. Section 5 presents the case study from the dairy industry to exemplify and evaluate the CT-based Industry 4.0 application development and presents the evaluation results. Finally, Section 6 presents the conclusion and future research directions.

## 2. Related Work

Traditionally, Industry 4.0 applications are developed by directly integrating the machines with the application via an existing IoT platform. However, with the emergence of Digital Twins (DTs), the DTs have also been utilized to develop Industry 4.0 applications. Existing research and commercially available IoT platforms have introduced a variety of DTs to represent industrial machines and to develop Industry 4.0 applications for managing and improving plant efficiency, product quality, and preventive maintenance. Section 2.1 briefly describes how these DTs are different and then reviews their applications and the provided support for Industry 4.0 application development. Section 2.2 reviews the traditional Industry 4.0 application development approach and the support provided by the existing IoT platforms for Industry 4.0 application development. Next, Section 2.3 reviews the existing cost models proposed and used to calculate the Industry 4.0 application development costs, and finally, Section 2.4 summarizes the review findings.

### 2.1. DTs and DT-Based Industry 4.0 Application Development

DT variants that have been proposed in the existing research include Digital Models (DMs), Digital Shadows (DSs), and Digital Twins (DTs). These variants differ in the level of data integration between the physical object and its corresponding virtual object (i.e., the digital representation) [22]. A DM is a virtual portrayal of a physical object with no automated data exchange between the physical object and the virtual object. In a DS there exists an automatic uni-directional dataflow from the physical object to the virtual object; thus, the virtual object reflects any changes to the state of the physical object. In DTs, there is a bi-directional data flow between the virtual and physical objects and the changes made to the DTs are reflected in the corresponding physical object and vice versa [10,22]. These digital representations have been proposed and are used for providing smart data-driven solutions to make improvements in manufacturing plants.

The DSs can collect and integrate data from the physical object and other relevant sources in real-time as they have an automated data flow from the physical to the virtual object. They can enable numerous applications to use these data to perform a comprehensive analysis on the corresponding machines or the manufacturing systems. To allow incident detection and the deciphering of the related operation context of the machining incident, a DS is proposed in [23]. The framework proposed for knowledge-based DSs allows the integration of data from diverse sources to create the DS, including from the machine tool itself and the smart sensing devices. Moreover, an ontology model is used to create the knowledge model of the proposed DS framework and the ontology support describing the detection elements, operational context, and incident types. The authors of [14] propose integrating the DS simulation model with the Manufacturing Execution System (MES) to create a DT. The MES integrated DT is used for decision making with the support of an underlying intelligence layer that hosts rules and the knowledge to choose among alternatives. Next, the DT is used to support the management of error states and to trigger disassembly processes that result from low assembly quality. In [24], a DS framework is proposed to enable real-time data collection and the integration of the maintenance, repair, and overhaul services of machine manufacturers. This includes the support of different kinds of maintenance activities including preventive and corrective maintenance. Moreover, the authors of [25] propose DSs to enable the efficient use of knowledge management systems to support single small batch production companies towards Industry 4.0. Riesener et al. has proposed a DS as an enabler for data analytics in product lifecycle management. The proposed model merges data from heterogeneous sources and uses mechanisms to choose the suitable data sources to obtain the required information [26]. While DSs allow information fusion from heterogeneous sources and can support the comprehensive analysis of machines and manufacturing systems, they do not support actuating and sending data to the machines.

The DTs have a bi-directional data flow between the physical and virtual objects and have the potential to support many facets of Industry 4.0 application development. To support the predictive maintenance of a flexible production system, Barthelmey et al. have proposed a dynamic DT [27]. The DT receives data in the AutomationML format, and the available analytical models are applied to the data to identify the predictive maintenance requirements. To support localized anomalous faults and also to infer the product quality of fused deposition modelling-based additive manufacturing printers, DTs are proposed in [15]. The work proposes an IoT-based methodology to build DTs using data from an indirect medium (i.e., retrofitted low-end sensors available in IoT devices) as the legacy manufacturing systems do not have built-in multi physics sensors by default. To support the condition monitoring of a CNC machining tool, Liu et al., have proposed a DT modelling framework [28]. In the proposed approach, the functions of the machining tool are provided as services to support manufacturers, enterprises, and operators by the DT. This enables a variety of applications to utilize the DT data. Moreover, Schroeder et al. [29] have proposed a data modelling mechanism for DTs of the industrial plant components using AutomationML. To support utilizing DT data by applications, the DT data are exposed to third-party applications via a middleware platform, using JSON/REST interfaces. However, the lack of the use of semantics can hinder the portability of the applications that utilize these data. To reduce the cost of programming and reconfiguring the robots that are used to improve the flexibility of production systems, Hoebert et al. have proposed DTs [30]. In this work, ontologies are used as a knowledge base to describe the robot and the related environment to support the automatic configuration of the DT and the robot. To advance the traditional CNC machine tools to Cyber-Physical Machine Tools (CPMT), Liu et al. have proposed a systematic development method. The CPMT encompasses a Machine Tool Cyber Twin (MTCT) that contains an information model of the machine tool, supports data fusion, and embeds intelligent algorithms in it. An MTConnect-based information modelling approach is used in this work to support the representation of the logical structure of the machine tool and its static and dynamic properties [17]. To support a variety of applications, Lu and Xu have proposed a resource virtualization framework for smart factories [31]. The proposed framework encompasses a methodology for DT creation that is based on the hierarchy of the DT, the information to be modelled, and the related modelling method. However, this requires a considerable amount of manual work. In [20], DTs are proposed for a production line to support the optimization of the production processes using OPC data. In the proposed DT, the sensor data from the sensors of the production line machines are collected to provide a visual representation. This simulation-based model is then used to identify and alerts about the deviations from the optimal scenarios. Moreover, DTs have gained a lot of attention from industries. General Electric (GE) has invented the DT of a wind farm, where the DTs are constantly updated based on the data collected from the control systems of wind turbines in a farm [13]. The system allows the running state of wind turbines to be monitored through the respective digital models. Moreover, GE developed a DT interface to manage multiple DTs at the same time, which displays the latest operating conditions of the wind turbines and control features that can be (re)configured to optimize the wind farm performances. The DTs have the potential to support different aspects of Industry 4.0 application development. However, despite their potential in many cases, DTs are proposed to support specific applications [15,27,28]. Moreover, the lack of semantically rich machine descriptions hinders the ability to use the DTs in application development [29]. In addition, existing DTs have a limited ability to support the simulating/emulating complex machines including their machine automation [17,27,29,30,31] or to allow switching between the physical machines and the simulators to support Industry 4.0 application testing [20,32].

Furthermore, open source and commercial IoT platforms exist that support DT development and their use. GE’s Predix platform allows the creation of DTs and the running of data analytics and monitoring [33]. Moreover, the Siemens MindSphere platform allows machines and physical infrastructure to be connected to a DT and allows data to be streamed from these machines to support the development of DT solutions [34]. Moreover, Azure DT is a PaaS solution by Microsoft for supporting DT development. Azure DT provides a Digital Twin Definition Language (DTDL) to create DT model definitions and provides APIs to interact with DTs [8]. Azure also supports sensor data simulations and provides services for querying and finding the deployed DTs. Moreover, Eclipse Ditto is an open-source framework built to support the development of the DTs of “things”. The domain model used by Ditto for modelling has the concepts “thing”, “access controllers”, and “features”. Ditto maintains the model of the device and updates the model with the last reported state of the IoT device and further provides services for using the sensing and actuation capabilities of the device via its DT. It also provides search functionality for the applications to search and find a DT that maps a given criterion [35]. We have further discussed and evaluated the use of such IoT platforms and frameworks for building DTs in [21]. Currently, limited research has been completed to support cost-efficient DT generation to support Industry 4.0 applications and most of these solutions that support DT development require a considerable amount of manual work [31].

### 2.2. Traditional Industry 4.0 Application Development

Traditionally, Industry 4.0 application development is completed by (directly) utilizing IoT platforms. The machines are integrated with the IoT platforms to develop the Industry 4.0 applications. Here we review the support provided by the IoT platforms to develop Industry 4.0 applications related to the identification of machine data, the integration with the Industry 4.0 applications, and Industry 4.0 application development and testing when using this approach. Currently, identifying the machine data that are required by an Industry 4.0 application is time-consuming due to the unavailability of standard and semantically rich machine descriptions. Most of the IoT platforms enable the use of simple key-value pair-based machine descriptions [36,37] or support the use of IoT platform-specific vocabularies [38] to describe the machines. These descriptions are often developed by application developers who do not have expert knowledge about complex machines; thus, they lack accuracy. Moreover, complex machines can have many sensors, actuators, and multiple machine automation that utilize the sensors and actuators. Simple key-value pair-based descriptions have a limited capability to describe the complex inter-relationships among these elements. Further, due to the lack of the use of rich semantics, such descriptions can be difficult to understand [5,6]. To support the modelling of machines and their data, ontology-based approaches have been proposed in the existing research work [39,40,41]. IoT platforms such as OpenIoT [42] allow the SSN [43] ontology-based integration of sensors and their data with the IoT platform. Moreover, the DataTweet [44] framework allows semantic descriptions of the machines to be utilized to integrate them with the framework. However, the utilized ontologies do not support the modelling of complex machines. The platforms such as FIWARE support the adoption of common data models to support the interoperability of the applications [45]. They also contain reusable data models for domains such as smart cities, smart health, and smart aeronautics [46]. However, these need to be expanded to support the context of manufacturing and the modelling of complex machines including their machine automation. Moreover, MindSphere [34] supports Semantic Data Interconnect (SDI) to support the use of ontologies to understand and maintain relationships among data from different sources such as manufacturing resource planning, IoT data lakes, etc. This aims to support the building of interoperable solutions. However, it is necessary to have ontology models that support the description of the machines and their data. This is not provided by the platform. IoT platforms such as SiteWhere [36] and DataTweet allow application development using the machines integrated with the platform and provide REST-based APIs to send/receive data to/from the machines [44,47]. Moreover, Azure IoT provides the Azure IoT Hub service to integrate the machine with the IoT platform and provides AMPQ-based endpoints to retrieve machine data for application development [37]. However, due to the lack of the use of semantics in data integration, such solutions can lead to vendor lockdown. Moreover, certain machine outputs may need to be translated related to the context of the new application before integration with the application. To support this kind of translation, Azure IoT provides Azure functions [8]. However, due to the lack of the use of semantics, the reusability of such data translators and the related programming effort can be limited. Existing IoT platforms facilitate services that support the development of applications such as data analysis support, stream processing services, etc. [34,37]. However, there is a lack of support for facilitating the testing of Industry 4.0 applications that utilize complex machines. IoT platforms such as OpenIoT have a limited ability to support the application testing of complex machines. Industrial solutions such as Azure IoT provide device simulation services and allow sensor data simulation [48]. However, if it is necessary to simulate the aspects of machine automation, they require a considerable amount of work to implement the simulators and code their behavior [9]. Further, this may require knowledge about the behavior of machine automation. Moreover, the application update and porting require similar issues relating to each application development activity that was described earlier to be solved. Furthermore, the unavailability of reusable data translators and their descriptions can further hinder the cost-efficient update and portability of an application.

### 2.3. Industry 4.0 Application Development Cost Modelling

Developing an Industry 4.0 application requires the identification of machine data needed by an application, integrating these with the application, developing the application functionality, and testing the application using machine simulators or emulators. Cost models exist that have been proposed to calculate general software development costs such as COCOMO [49]. Moreover, to measure the reusability and portability of the software, evaluation formulas based on metrics such as man-hours, lines of code, are available [50,51]. However, these cost models do not capture the Industry 4.0 application development costs at a fine-grained level considering the application interactions with the machines and the application development activities, including an understanding of the machines and integrating their data.

### 2.4. Summary

The traditional approach towards Industry 4.0 application development is impeded by the lack of accurate and comprehensive machine descriptions, inefficient machine data integration approaches with the Industry 4.0 application development, and limited support for application testing. The existing research and commercially available IoT platforms have introduced a variety of DTs to represent industrial machines and to support the development of Industry 4.0 applications. However, there are limitations in existing DTs with respect to their ability to support Industry 4.0 application development, testing, update, and porting. Existing DTs have a limited ability to simulate and/or emulate the aspects of complex machines, including their machine automation controllers, and they do not support switching between physical machines and their respective simulators to support application testing. Moreover, some of them are application-oriented and are unable to support a variety of Industry 4.0 applications. Furthermore, cost-efficient DT development needs to be supported to enable Industry 4.0 application development using DTs; however, this has not been sufficiently explored in the research. In addition, it is important to support the updating of the Industry 4.0 applications to facilitate the change of machines and porting them across manufacturing plants; this has not been explored in existing DT solutions. To overcome the above limitations and to enable cost-efficient Industry 4.0 application development, this paper proposes CTs that are an extension to DTs. A CT can support a variety of applications, has a semantic description of the machine that it represents, and includes capabilities to support cost-efficient application testing, updating, and porting. The CTs are introduced in Section 3 and CT-based Industry 4.0 application development is discussed in Section 4.

Moreover, there is a lack of cost models for Industry 4.0 application cost/benefit analysis and the available cost models only allow the modelling of general application development costs. To address this, this paper also proposes a cost model for calculating the Industry 4.0 application development costs and this is introduced in Section 4.2.

## 3. Cyber Twins (CTs)

CTs are digital representations of physical machines. We note here that the paper covers only the area of Industry 4.0 applications (e.g., preventive maintenance, product quality improvement) and in Industry 4.0, the main physical entities are industrial machines. Thus, CTs provide digital representations to them to support Industry 4.0 application development. The CTs support bi-directional communication between the physical machine and the CT. A CT reflects the state of the physical machine, and any changes made to the state of the CT are reflected on the physical machine, similar to existing DTs. Moreover, CTs closely reflect the behavior of the physical machine by incorporating an emulator/simulator of the machine. This can be a near real-time reflection of the behavior of the physical machine if the provided simulators can be configured to accept real-time inputs from the machine, or it can be a close reflection of the expected behavior of the machine if the simulators are provided with historical data from the machine, or if a machine emulator is used. Furthermore, the proposed CTs extend the existing DTs by addressing some of the limitations in existing DTs to support Industry 4.0 application development, testing, update, and porting. CTs can be cost-efficiently auto-generated by using the machine semantic descriptions and contain semantic descriptions of the machines and the CTs themselves. In addition, CTs can be easily updated to accommodate machine upgrades or replacements. Further, a CT can support a variety of Industry 4.0 applications and provides the following services to support their development:

- Query the semantic description of the machine that is represented by the CT;
- Communicate with the machine including obtaining the data produced by the machine, applying the machine settings, and sending other inputs to the machine;
- Interact with the machine emulator or simulator that is incorporated in the CT to support application testing.

In the following sections, we present a brief overview of the CT ontology that is used as a basis for capturing the semantic description of any specific machine and explain CT generation via a CT management framework, which was introduced in [21]. Moreover, we briefly explain how the simulators in CTs can support Industry 4.0 application testing.

### 3.1. CT Ontology for Describing Complex Industrial Machines

**Figure 1:**
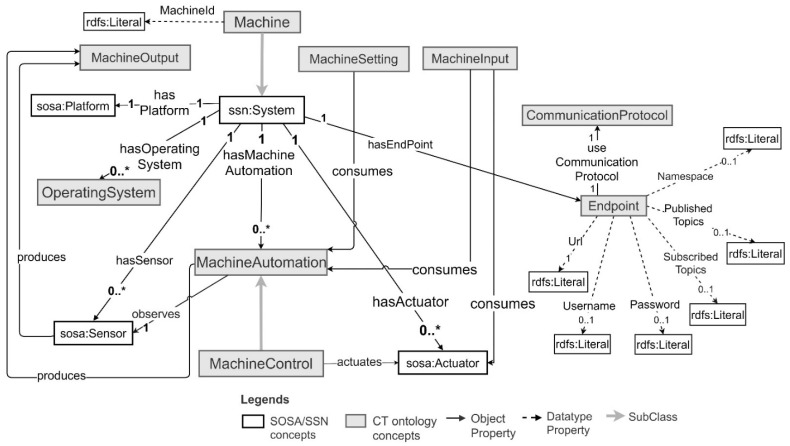
The CT ontology for modelling machines. The CT ontology reuses the sensor, actuator, system, and platform concepts from SOSA/SSN ontology and introduces the machine, operating system, machine automation, machine control, communication protocol, and endpoint concepts to model the machines and elements of a machine. It also supports the modelling of machine data using machine inputs, machine outputs, and machine settings.

The concepts and relationships provided in the CT ontology to capture machines and their data are depicted in Figure 1. The ontology can be used to model simple machines, which contain a single sensor and an actuator, or complex machines with many sensors, actuators, and machine automation. It contains Communication Protocol and Endpoint concepts to be able to capture the connectivity details of a machine. It reuses the concepts such as Sensor, Actuator, System, and Platform from existing SSN [43]/SOSA [52] ontologies and these are prefixed using “sosa” and “ssn” in Figure 1. An example of CT ontology-based machine modelling is presented in Section 5.1.

### 3.2. CT Management Framework

**Figure 2:**
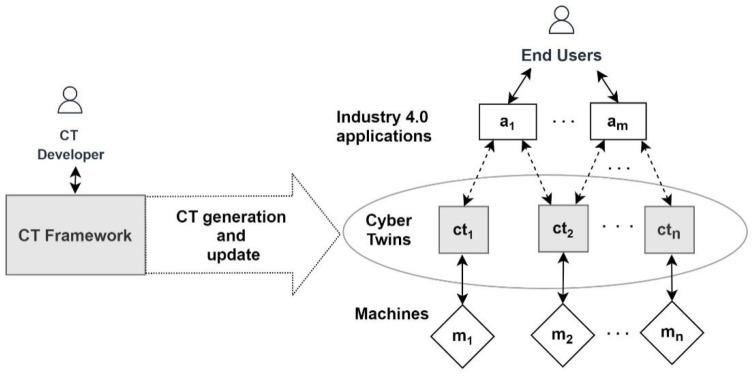
The CT management framework. The framework enables the CT developers to generate and update the CTs of the machines. The Industry 4.0 applications utilize the machines via their corresponding CTs, and end-users interact with these Industry 4.0 applications.

The framework provides services for the CT developers to generate the CTs for the machines and updates them based on the machine semantic descriptions. Figure 2 illustrates the framework services.

To generate a CT for a machine, a CT developer first needs to model the machine using the CT ontology. A modelling tool such as Protégé [53] can be used for modelling purposes. Then the semantic description of the machine and the information that needs to be associated with the CT needs to be provided to the CT generation service. This service accepts the machine’s semantic description, CT ID, and context information relating to the CT as its inputs. The context information is optional and can include a description of the environment (e.g., the plant where the machine is located), as well as the latitude and the longitude of the environment’s location. In response to the service request, the service generates the CT for the machine and deploys it into the CT deployment environment integrated with the framework.

**Figure 3:**
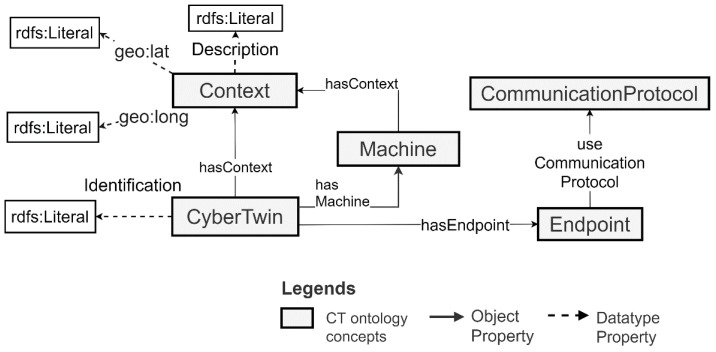
The ontology concepts that are used for generating the semantic descriptions of the CTs. The ontology concepts include Cyber Twin, machine, context, endpoint, and the communication protocol.

To support CT-based Industry 4.0 application development, the CT generation service also generates and associates a semantic description of the CT with the generated CTs. This description contains the CT ID, machine ID, the context, and the endpoints required to connect with the CT. The ontology concepts that are used for generating this description are shown in Figure 3.

The context is used to model the information provided by the CT developer to the CT generation service. It has Datatype properties to model the longitude (geo:long), latitude (geo:lat) and description (Description) of the CT. Moreover, a CT can have multiple endpoints that support different Communication Protocols for the sending/receiving of machine data and the querying of semantic descriptions. Section 5.2 includes an example of a specific CT semantic description. The CTs generated for the machines become available to the application developers via a web interface. This interface allows the application developers to apply filter criteria to find the CTs and query the machine semantic descriptions via their corresponding CTs to support the integration of the machines with the applications.

The CT framework allows the CTs to be updated to support machine replacement. More specifically, updating a CT to connect to another machine involves providing the CT ID and the machine description of the new machine as an input to the CT update service provided by the CT management framework. When the respective service receives this request, it will update the selected CT and connect it to the new machine. Here, a brief overview of the generation and updating of the CT was provided for the comprehensiveness of the paper, and the reader is referred to [21] for a detailed discussion on the topic. Please note that this paper only focuses on the use of the generated CTs to develop the applications.

### 3.3. Simulators in CTs and Their Support for Testing

Industry 4.0 application testing is an important aspect of Industry 4.0 application development. Generally, machine simulators are available for many industrial machines and testing typically involves using these simulators [54,55]. The proposed CTs incorporate the physical machine as well as simulators of the corresponding machines to support application testing. CTs allow a choice between the simulator or emulator of the machine they represent as is needed to carry out application testing. This approach allows CTs to support Industry 4.0 application testing and improvement. Later in the paper, in Section 5.2.2, we describe the specific simulators used for testing the application described in Section 5.

Next, we describe the Industry 4.0 application development using the CTs and the proposed cost model for modelling Industry 4.0 application development cost.

## 4. CT-Based Industry 4.0 Application Development and Costing

CT-based Industry 4.0 application development involves selecting the CTs that are needed by the application, integrating the CT and the machine data they provide with the application, testing the application using CT-provided machine simulators, and deploying the application with the CTs set to use the actual machines. Section 4.1 presents CT-based application development and the roles of users that develop and use CTs. Section 4.2 introduces a cost model for Industry 4.0 applications that will be used later in this paper to show the benefits of CT-based application development.

### 4.1. CT-Based Industry 4.0 Application Development: Activities and Roles

CT-based Industry 4.0 application development also includes application updates and application porting and involves two user roles: CT developer and application developer. The CT developer generates the CTs for the machines and updates the CTs. CT generation is only required if a machine that needs to be used by an application does not already have a CT. Thus, CT generation only needs to be performed once and once generated, a CT can be used/reused by any Industry 4.0 application that needs to use the corresponding machine. Moreover, a CT can be updated by the CT developer as required to support its usage in application development. The application developer uses CTs to develop the Industry 4.0 applications.

In the following sections, we discuss the activities involved in (a) developing a new CT-based Industry 4.0 application, (b) updating an existing CT-based Industry 4.0 application to a change or an upgrade of a machine and (c) porting an existing CT-based Industry 4.0 application to a different environment (e.g., a plant or a farm).

#### 4.1.1. Developing a New CT-Based Industry 4.0 Application

**Figure 4:**
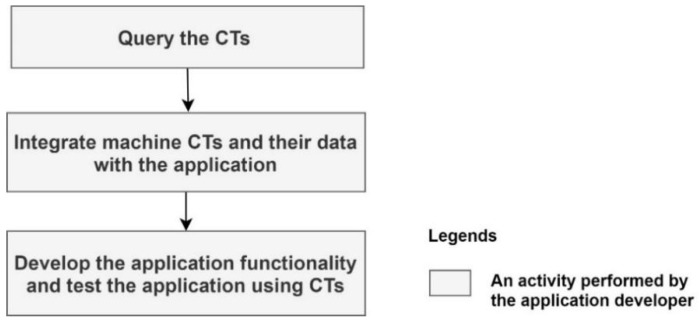
New CT-based Industry 4.0 application development, related activities, and user roles.

For new application development, the application developer needs to query the machine description via the CT and identify the machine data needed by the application. If the machine produces the data that map the application requirements, then the data need to be integrated with the application via the CT. Finally, it requires the development of the application functionality and the testing of the application using the CTs. These activities are further illustrated in Figure 4.

#### 4.1.2. Updating an Existing CT-Based Industry 4.0 Application

**Figure 5:**
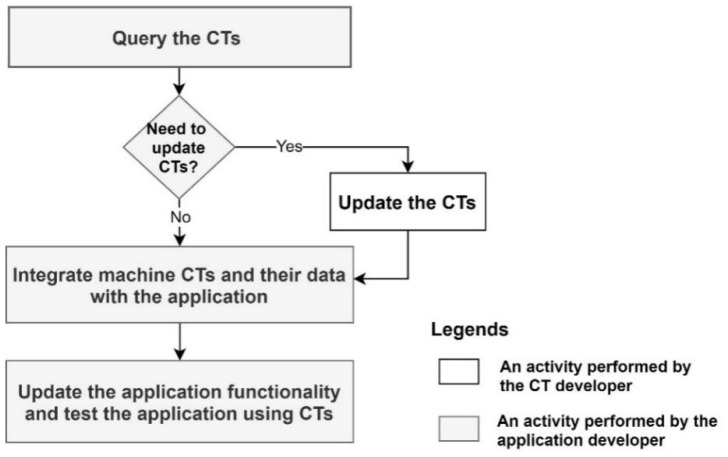
CT-based Industry 4.0 application update, related activities, and user roles.

If a machine used by an application is upgraded, changed, or replaced, it may require an update to the Industry 4.0 application that uses the machine via the CT. If the new machine has its own CT, this requires querying the CT of the new machine, integrating its data with the application, and finally (if required) updating the application functionality and testing it. If the new machine produces different data, the CT of the new machine needs to be updated by applying a data translator and its data need to be integrated with the application. This is further illustrated in Figure 5.

Note that if the CT of the old machine is updated to be connected to the new machine, the application code does not need to be modified unless it requires data translation.

#### 4.1.3. Porting an Existing CT-Based Industry 4.0 Application

If an application needs to be ported to a different environment (e.g., to a different plant) it may require changes to the application. If machines in the new environment have CTs, the application developer needs to query the machine descriptions, integrate the new CTs with the application and (if required) update the application functionality and test the application. Moreover, the CTs of the new machines can be updated as necessary by applying the data translators. These activities are the same as those depicted in Figure 5. In the case that the new machines do not have CTs, the CTs need to be generated.

### 4.2. A Cost Model for Industry 4.0 Application Development

**Figure 6:**
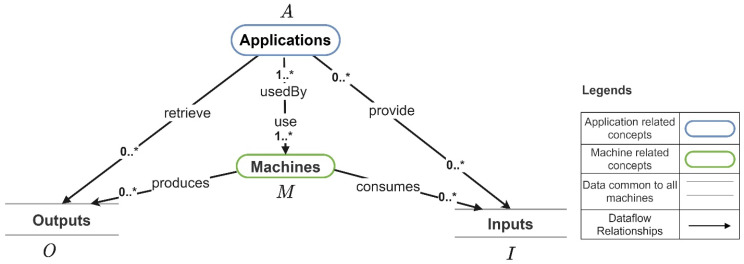
Industry 4.0 applications utilizing machines. The applications retrieve outputs and provide inputs, and the machines consume the inputs and provide outputs.

In this section, we introduce a cost model for the Industry 4.0 application that allows us to compare the cost of CT-based application development with other application development alternatives. The proposed cost model considers all application development activities that go into Industry 4.0 application development, so that CT-based application development can be compared to IoT-platform-based application development. More specifically, the proposed cost model considers the costs of the following for each Industry 4.0 application: (a) identifying the machine data needed by the application, (b) integrating machines and their data with the application and (c) developing the application functionality and testing the application. Before introducing the formulas for calculating the application development cost, we first present how the machines are used by Industry 4.0 applications based on the data flow interactions between them. This is depicted in Figure 6.

**Figure 7:**
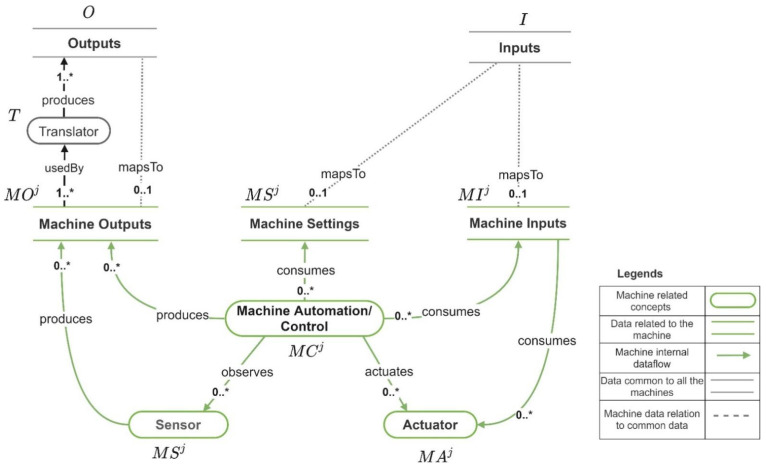
Internal elements of machine $m_{j}$, the machine data produced and consumed by $m_{j}$, data translators applied to $m_{j}$, inputs, and outputs. Machine $m_{j}$ consumes machine settings and machine inputs and produces machine outputs.

Figure 7 depicts the internal data flow of a machine $m_{j}$, including the machine outputs, machine inputs, and machine settings and Figure 6 depicts how they are mapped to set of outputs (O) and set of inputs (I). The set of machine outputs produced by $m_{j}$ is $MO^{j}$, the set of machine inputs consumed by $m_{j}$ is $MI^{j}$, and the set of machine settings consumed by $m_{j}$ is $MST^{j}$. For each machine $m_{j}\;\left(m_{j}\in M\right)$, $MI^{j}\subset \;O$, and $MO^{j}\;\subset \;I\;\mathrm{and}\;MST^{j}\subset \;I$. In a case where a machine output cannot be directly mapped to an input that needs to be retrieved by an application, the utilization of a data translator (a program that implements the functionality for converting the machine outputs to the required application input) is required. The set of data translators that allow the translation machine outputs are denoted by T. $MS^{j}$ and $MA^{j}$ denote the set of sensors and the set of actuators in the machine $m_{j}$. $MC^{j}$ denotes the set of machine automation/machine controls in $m_{j}$. If $m_{j}$ is a simple machine, $\left|MC^{j}\right|=0$. If $m_{j}$ is a complex machine $\left|MC^{j}\right|>0$. Next, we present the formula for calculating the total cost of developing an Industry 4.0 application.

If $Cost_{identify}\left(M^{l},a_{l}\right)$ denotes the costs of identifying the data produced by $M^{l}$ that needs to be used by the application $a_{l}$, $Cost_{int}\left(M^{l},a_{l}\right)$ denotes the cost of integrating $M^{l}$ and their data with $a_{l}$, and $Cost_{dt}\left(M^{l},a_{l}\right)$ denotes the cost of developing the application functionality and testing $a_{l}$ using $M^{l}$, the total cost of developing $a_{l}$, $Cost_{Total}\left(a_{l}\right)$, is,

(1) $$Cost_{Total}\left(a_{l}\right)=Cost_{identify}\left(M^{l},a_{l}\right)+Cost_{int}\left(M^{l},a_{l}\right)+Cost_{dt}\left(M^{l},a_{l}\right)$$

A summary of the notations introduced earlier is listed in Table 1. Next, we describe how the $Cost_{identify}\left(M^{l},a_{l}\right)$, $Cost_{int}\left(M^{l},a_{l}\right),$ and $Cost_{dt}\left(M^{l},a_{l}\right)$ are calculated and the metrics that can be used in the cost calculations.

#### 4.2.1. Cost of Identifying the Machine Data Required by the Application

The total cost of identifying all the machine data that need to be utilized by an application is the summation of the costs of identifying the data needed by an application from each machine utilized by the application. If $C_{identify}\left(m_{j},a_{l}\right)$ denotes the cost of identifying data required by $a_{l}$ from $m_{j}$, $Cost_{identify}\left(M^{l},a_{l}\right)$ is,

(2) $$Cost_{identify}\left(M^{l},a_{l}\right)=\sum_{\forall m_{j}\in M^{l}}C_{identify}\left(m_{j},a_{l}\right)\;$$

Here, $C_{identify}\left(m_{j},a_{l}\right)$ includes the costs of identifying the machine outputs directly consumed by the application $(MO^{j}\cap O^{l,j}$), the machine inputs $\left(MI^{j}\cap \;I^{l,j}\right),$ and the machine settings $\left(MST^{j}\cap I^{l,j}\right)$ needed by the application. If $C_{identify}$(y) denotes the cost of identifying an individual element y, where $y\in \left(MO^{j}\cap O^{l,j}\right)\;OR\;y\in \left(MI^{j}\cap I^{l,j}\right)\;OR\;y\in \;\left(MST^{j}\cap I^{l,j}\right)$,

(3) $$\begin{array}{c} C_{identify}\left(m_{j},a_{l}\right)\\ =\sum_{\forall p\in \left(MO^{j}\cap O^{l,j}\right)}C_{identify}\left(p\right)+\sum_{\forall r\in \left(MI^{j}\cap I^{l,j}\right)}C_{identify}\left(r\right)\\ +\sum_{\forall \;t\;\in \;\left(MST^{j}\;\cap \;I^{l,j}\right)}C_{identify}\left(t\right) \end{array}$$

Here, $C_{identify}$(y) includes the costs of querying and applying the filter criteria (or executing commands) to identify y. If $C_{query}$(y) denotes the cost of querying the identification of element y and $C_{config}$(y) denotes the cost of applying configurations (e.g., applying filter criteria or commands) to identify y,

(4) $$C_{identify}(y)=C_{query}(y)+C_{config}(y)$$

It is possible to use the Number of Configurations (NoCs) and the Number of Queries (NoQs) as the metrics for calculating these costs. We define $k_{1}$ to be the querying cost factor (e.g., cost of writing a query) and $k_{2}$ to be the configuring cost factor (e.g., cost of applying filter criteria or a command). If $w_{1}$ is the number of queries executed for identifying y and $w_{2}$ is the number of configuration parameters (or commands) applied for identifying y, by applying these to Equation (4) we obtain,

(5) $$C_{identify}\left(y\right)=k_{1}w_{1}+k_{2}w_{2}$$

$Cost_{identify}\left(M^{l},a_{l}\right)$ can be calculated by calculating $C_{identify}\left(m_{j},a_{l}\right)$ for each machine $m_{j}$ using Equations (4) and (5).

#### 4.2.2. Cost of Integrating Machines and Machine Data with the Application

The total cost of integrating all machines utilized by an application and the related data is a summation of the costs of integrating each machine and its related data with the application. If $C_{int}\left(m_{j},a_{l}\right)$ denotes the cost of integrating machine $m_{j}$ and its data to use in $a_{l}$,

(6) $$Cost_{int}\left(M^{l},a_{l}\right)=\underset{\forall m_{j}\in \;M^{l}}{\sum }C_{int}\left(m_{j},a_{l}\right)\;$$

Here, $C_{int}\left(m_{j},a_{l}\right)$ includes the costs of integrating $m_{j}$ and its data and integrating the related data translators with the application. If $C_{int}$(y) denotes the cost of integrating an individual element y, where $y\in \left\{m_{j}\right\}\;OR\;y\in \;T^{l,j}$, $C_{int}$($m_{j},a_{l}$) is,

(7) $$C_{int}\left(m_{j},a_{l}\right)=C_{int}\left(m_{j}\right)+\underset{\forall \;q\;\in \;\left(T^{l,j}\right)}{\sum }C_{int}\left(q\right)\;$$

Integrating a machine and its data or the related data translators with the application requires the configurations and/or writing code needed for integration to be set. If $C_{config}$(y) denotes the cost of setting configurations to integrate y and $C_{code}$(y) denotes the cost of coding for integrating y,

(8) $$C_{int}(y)=C_{config}(y)+C_{code}\left(y\right)$$

We use Source Lines of Code (SLoC) and NoCs as the metrics to calculate the costs. If $x_{1}$ is the NoCs applied (or executed) for integrating y and $x_{2}$ is the SLoC added or modified for integrating y, $k_{2}$ is the configuring cost factor, and $k_{3}$ is the coding cost factor, by applying these to (8) we obtain,

(9) $$C_{int}\left(y\right)=k_{2}x_{1}+k_{3}x_{2}$$

Subsequently, $Cost_{int}\left(M^{l},a_{l}\right)$ can be calculated by calculating $C_{int}\left(m_{j},a_{l}\right)$ for each machine $m_{j}$ using Equations (8) and (9).

#### 4.2.3. Cost of Developing the Application Functionality and Testing the Application

The cost of developing the application functionality and testing it using the machine simulators/emulators is the summation of the costs of the coding required for developing the application functionality and the cost of testing it using the machine simulators/emulators. If $C_{code}\left(a_{l}\right)$ denotes the coding cost of developing $a_{l}$ and $C_{test}\left(M^{l},a_{l}\right)$ denotes the cost involved in testing $a_{l}$, by using the simulators/emulators of $M^{l}$,

(10) $$Cost_{dt}\left(M^{l},a_{l}\right)=C_{code}\left(a_{l}\right)+C_{test}\left(M^{l},a_{l}\right)\;$$

It is possible to use SLoC as the metric for calculating $C_{code}\left(a_{l}\right)$. If $y_{1}$ is the SLoC added or modified for implementing the application functionality,

(11) $$C_{code}\left(a_{l}\right)=k_{3}y_{1}$$

$C_{test}\left(M^{l},a_{l}\right)$ is the summation of the costs of simulating/emulating each machine used by the application $a_{l}$ and the cost of writing code for testing the application $a_{l}$. If $C_{sim}\left(m_{j}\right)$ denotes the cost of simulating/emulating an individual machine, $m_{j}$ and $C_{test\_code}\left(a_{l}\right)$ denotes the cost of writing code to test $a_{l}$,

(12) $$C_{test}\left(M^{l},a_{l}\right)=C_{test\_code}\left(a_{l}\right)+\underset{\forall m_{j}\in M^{l}}{\sum }C_{sim}\left(m_{j},a_{l}\right)$$

We use SLoC as the metric for calculating $C_{test\_code}\left(a_{l}\right)$. If $y_{2}$ is the SLoC added or modified for testing $a_{l}$,

(13) $$C_{test\_code}\left(a_{l}\right)=k_{3}y_{2}$$

$C_{sim}\left(m_{j},a_{l}\right)$ includes the cost of developing machine simulators/emulators and the cost of integrating them with the application. If $C_{sim\_dev}\left(m_{j},a_{l}\right)$ denotes the cost of developing a simulator/emulator of $m_{j}$ to test $a_{l}$ and $C_{sim\_int}\left(m_{j},a_{l}\right)$ denotes the cost of integrating the simulator/emulator of $m_{j}$ to test $a_{l}$,

(14) $$C_{sim}\left(m_{j},a_{l}\right)=C_{sim\_dev}\left(m_{j},a_{l}\right)+C_{sim\_int}\left(m_{j},a_{l}\right)\;$$

As before, we use SLoC as the metric for calculating both $C_{sim\_dev}\left(m_{j},a_{l}\right)$ and $C_{int\_sim}\left(m_{j},a_{l}\right)$. If $y_{3}$ is the SLoC added or modified for developing the simulator of $m_{j}$ for $a_{l}$, and $y_{4}$ is the SLoC added or modified for integrating the simulator of $m_{j}$ with $a_{l}$,

(15) $$C_{sim}\left(m_{j},a_{l}\right)=k_{3}y_{3}+k_{3}y_{4}$$

It is possible to use Equation (13)–(15) for calculating $C_{test}\left(M^{l},a_{l}\right)$ in (10) and use (11) to calculate $C_{code}\left(a_{l}\right)$ in (10).

Finally the calculated values for $Cost_{identify}\left(M^{l},a_{l}\right)$,$\;Cost_{int}\left(M^{l},a_{l}\right)$ and $Cost_{dt}\left(M^{l},a_{l}\right)$ can then be applied in Equation (1) to find $Cost_{Total}\left(a_{l}\right)$.

Next, we introduce two formulas for assessing the adaptability of an existing Industry 4.0 application to a change of a machine (the degree of adaptability) and the portability of an existing Industry 4.0 application to a different environment (the degree of portability).

#### 4.2.4. Degree of Adaptability of an Industry 4.0 Application

The degree of adaptability of an application is formulated based on the cost of updating an existing application to the change of machines and the cost of redeveloping the same application to use with the changed machines. $C_{dev}\left(a_{l},\;M^{l}\right)$ denotes the cost of developing an Industry 4.0 application $a_{l}$ by using the set of machines $M^{l}.\;C_{rdev}\left(a_{l},\;M^{{}^{\prime }l}\right)$ denotes the cost of redeveloping $a_{l}$ using the set of machines $M^{{}^{\prime }l}$. If X is the set of changed or replaced machines in $M^{l}$ and Y is the set of replacements, the updated set of machines $M^{{}^{\prime }l}=\{M^{l}\backslash \;X\}\;\cup Y$ and |X |=|Y|. $C_{update}\left(a_{l},\;M^{{}^{\prime }l}\right)$ denotes the cost of updating $a_{l}$ to be used with $M^{{}^{\prime }l}$. Based on these, the degree of adaptability of $a_{l}$, $DA\left(a_{l}\right)$ is,

(16) $$DA\left(a_{l}\right)=1-\left(C_{update}\left(a_{l},\;M^{{}^{\prime }l}\right)/C_{rdev}\left(a_{l},\;M^{{}^{\prime }l}\right)\right)$$

If $DA\left(a_{l}\right)$ is a high positive value, the application is easy to update and the cost of updating the application is low compared to the cost of redeveloping the application and vice versa. $C_{update}\left(a_{l},\;M^{{}^{\prime }l}\right)$ can be calculated using (1) as the basis, considering the related cost of identifying the machine data needed by the application from the changed machines, the cost of integrating them with the application, and the cost of doing any modification to the application code and testing the application. $C_{rdev}\left(a_{l},\;M^{{}^{\prime }l}\right)$ can also be calculated using Equation (1) as the basis, considering the related cost of identifying the machine data needed by the application from $M^{{}^{\prime }l}$, the costs of data integration, and the cost of application functionality development and testing.

#### 4.2.5. Degree of Portability of an Industry 4.0 Application

We have adopted the formula proposed by Mooney [50] to measure the degree of portability for evaluating the degree of portability of Industry 4.0 applications across environments. We denote an environment $p_{i}$, an example of which is a manufacturing plant or even a combination of multiple plants an application uses. If $C_{dev}\left(a_{l},\;p_{1}\right)$ denotes the cost of developing an Industry 4.0 application $a_{l}$ for environment $p_{1}$, $C_{rdev}\left(a_{l},\;p_{2}\right)$ denotes the cost of redeveloping the same Industry 4.0 application for a new environment $p_{2}$ and $C_{port}\left(a_{l},\;p_{2}\right)$ denotes the cost of porting the existing application $a_{l}$ to $p_{2}$, $DP\left(a_{l}\right)$ the degree of portability of $a_{l}$ is,

(17) $$DP\left(a_{l}\right)=1-\left(C_{port}\left(a_{l},\;p_{2}\right)/C_{rdev}\left(a_{l},\;p_{2}\right)\right)$$

If $DP\left(a_{l}\right)$ is a high positive value, the application is highly portable and the cost of porting the application is low compared to the cost of redeveloping the application and vice versa. $C_{port}\left(a_{l},\;p_{2}\right)$ can be calculated using Equation (1) as the basis, considering the related cost of identifying the machine data needed by the application from the machines in $p_{2}$, the cost of integrating them with the application, and the cost of doing any modification to the application code and testing the application. $C_{rdev}\left(a_{l},\;M^{{}^{\prime }l}\right)$ can also be calculated using Equation (1) as the basis, considering the related cost of identifying the machine data needed by the application from $M^{{}^{\prime }l}$, the related data integration costs, and the application functionality development and testing costs for $p_{2}$.

Next, we present a case study from the dairy industry to exemplify CT-based Industry 4.0 application development and to evaluate the CT-based Industry 4.0 application development using the proposed cost model.

## 5. Experimental Evaluation of a CT-Based Industry 4.0 Application

Here we first explain the milk pickup process from the dairy industry that involves milk tank and pickup truck machines and the milk pickup monitoring application. Next, in Section 5.1 we present the related CT ontology-based models of the machines used in the application. Section 5.2 presents the CTs generated for these machines and Section 5.3 discusses the CT-based milk pickup monitoring application development. Section 5.4 presents an evaluation that compares the costs of the development of this Industry 4.0 application using CTs with the alternative of using an IoT platform. Section 5.5 discusses the evaluation results.

**Figure 8:**
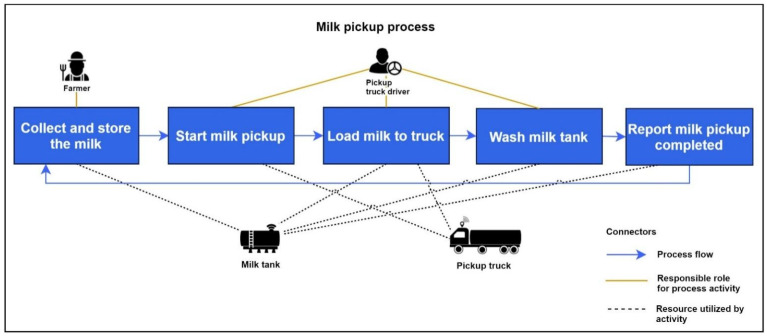
The milk pickup process, activities, responsible roles, and utilized resources. The milk pickup process utilizes milk tank and pickup truck machines as resources and the farmer and pickup truck driver are the responsible user roles.

In dairy farms, milk is collected by the farmers and stored in milk tanks until a pickup truck arrives. When the pickup truck arrives at the milk farm, milk pickup begins, and the pickup truck driver loads the milk from the tank to the pickup truck. After the milk loading is completed, the pickup truck driver initiates the milk tank wash. Following the milk tank wash, the milk tank reports the milk pickup as complete. An overview of this milk pickup process, the roles that are responsible for each of the processing activity, and the resources utilized by each activity are illustrated in Figure 8. The milk pickup monitoring application enables milk processors (who produce products from milk) to monitor the milk stored in tanks in an environment (e.g., a farm), including temperature and quantity, and to receive milk pickup events from the milk tank to be able to make process planning and forecasting decisions.

Next, we present the CT ontology-based model of these machines.

### 5.1. CT Ontology-Based Models of the Pickup Truck and the Milk Tank Machines

As explained earlier in Section 3, CTs aim to support the development of Industry 4.0 applications that utilize industrial machines. A CT of an industrial machine includes the corresponding sensors and actuators of the industrial machine, which are needed by the application that the CT is designed to support. For example, here the CT of the pickup truck machine in the use case is represented by the capture of the truck location data from its GPS sensors and a few other aspects that are needed for the milk monitoring application. However, note that the semantic representation of the Cyber Twin of the pickup truck can be modelled to include additional onboard sensors (e.g., acceleration sensors, airflow sensors, fuel sensors), actuators (e.g., fuel pump, control relays), and built-in automation (e.g., engine control unit) if these are required to support additional applications (e.g., diagnostics and preventive maintenance).

**Figure 9:**
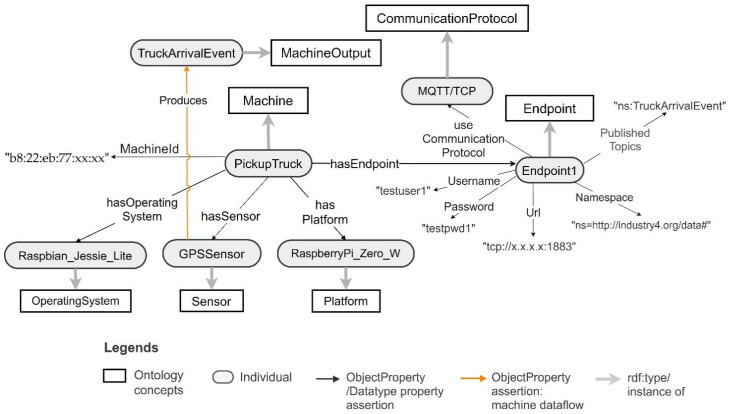
CT ontology-based model of the pickup truck. The model includes a GPS sensor, a Raspberry PI hardware platform, and a Raspbian operating system. The machine produces truck arrival events as machine outputs and has an endpoint that supports MQTT/TCP communication protocol.

The pickup truck machine has a GPS sensor connected to a Raspberry Pi Zero W and communicates over the MQTT communication protocol. It produces the truck arrival events based on GPS location data as the machine output. By using the CT ontology, the pickup truck can be modelled as an instance of a Machine. The GPS sensor can be modelled as a sosa:Sensor and the Raspberry Pi Zero W and the Raspbian operating system can be modelled as a sosa:Platform and OperatingSystem, respectively. Moreover, the machine’s communication interface can be modelled as an Endpoint and the related communication protocol can be modelled as a CommunicationProtocol. The truck arrival event can be modelled as a MachineOutput. Moreover, the machine has hardware identification that can be described using the Datatype Property MachineId. Figure 9 depicts the ontology model of a pickup truck with hardware identification “b8:22:eb:77:xx:xx”.

**Figure 10:**
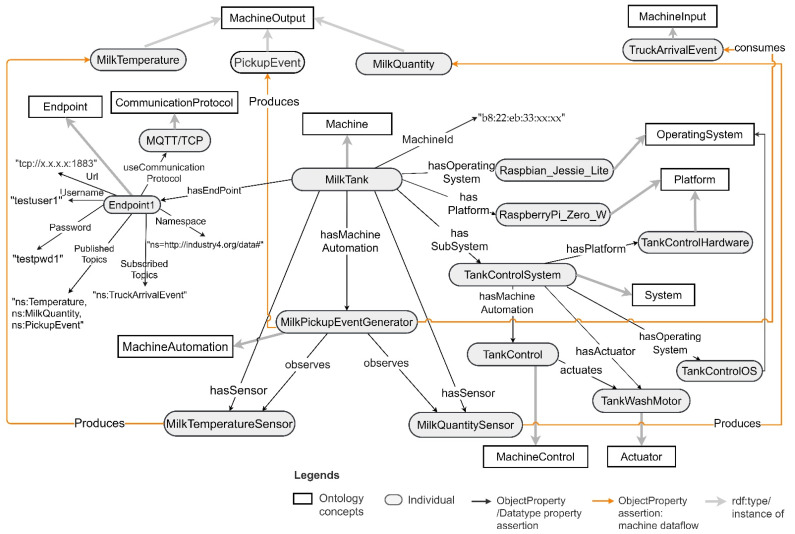
CT ontology-based model of the milk tank. The model has milk temperature and milk quantity sensors, a tank wash motor actuator, a pickup event generator machine automation, and built-in machine control that actuates the tank wash motor actuator. The machine produces milk temperature, milk quantity, and pickup events as machine outputs and consumes truck arrival events as machine inputs.

A milk tank is a complex machine. It has multiple sensors observing milk temperature, milk quantity, and tank wash motor actions connected to Raspberry Pi zero W hardware platform. Moreover, it has an automation program that observes the milk sensor data, milk tank wash motor actions, and truck arrival events, and generates milk pickup events. The machine also includes a built-in tank control that actuates the tank wash motor. The pickup event generator produces milk temperature, milk quantity, and pickup events as machine outputs and consumes truck arrival events as machine inputs. Using the CT ontology, a milk tank can be modelled as an instance of a Machine. Its sensors, actuators, platform, and operating system can be modelled as sosa:Sensor, sosa:Actuator, sosa:Platform, and OperatingSystem, respectively. The pickup event generator program can be modelled as MachineAutomation and the milk tank’s built-in control system can be modelled as an ssn:System. The tank control can be modelled as a MachineControl as it actuates the tank wash motor based on its control logic implementation. Then it can be connected to the milk tank machine as a subsystem via ssn:hasSubSystem object property. Moreover, similar to the truck, the machine hardware identification for the milk tank can be described using the DatatypeProperty MachineId. The milk tank model is depicted in Figure 10 for a milk tank with hardware identification “b8:22:eb:33:xx:xx”.

Next, we present the CTs generated for the pickup truck and the milk tank using the semantic description of the machines.

### 5.2. CTs of the Pickup Truck and the Milk Tank

The CTs of the machines incorporate CT descriptions as well as machine simulators. Here we present the CT descriptions and the used machine simulators for the pickup truck and the milk tank.

#### 5.2.1. CT Descriptions of the Milk Tank and Pickup Truck Machines

**Figure 11:**
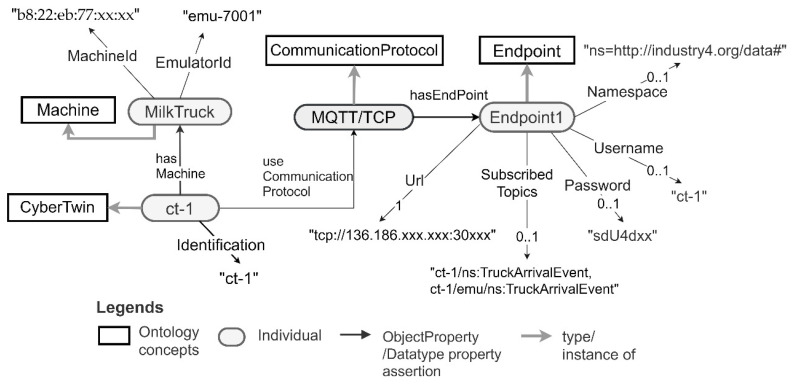
Pickup truck CT description. The CT description includes the machine ID, emulator ID, the identification of the Cyber Twin, the communication protocol, and the endpoint information provided by the CT to the application.

**Figure 12:**
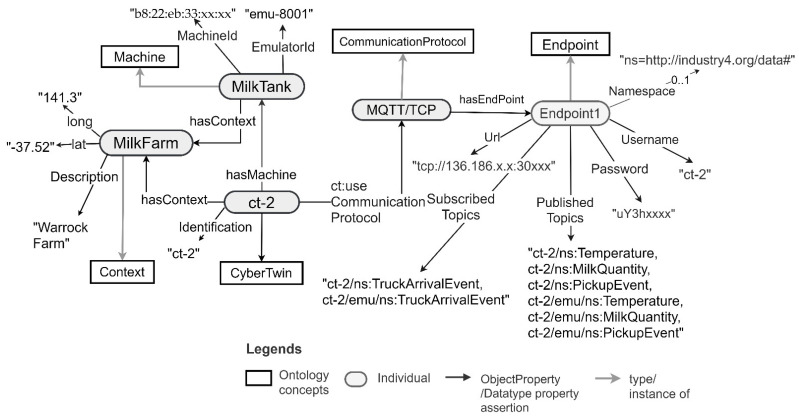
Milk tank CT description. The CT description includes the machine ID, the emulator ID, the identification of the Cyber Twin, the context related to the Cyber Twin and the machine, the communication protocol, and the endpoint information provided by the CT to the application.

The semantic descriptions of the CTs generated to support the integration of the pickup truck machine and its data are depicted in Figure 11. The same descriptions for the milk tank are depicted in Figure 12. Note that these are generated by the CT management framework when it generates the CTs for these machines. The framework uses the ontology concepts presented in Section 3.2. to generate these descriptions.

As depicted in Figure 10, the pickup truck CT has CT ID ct-1. It connects to the machine with machine ID “b8:22:eb:77:xx:xx” and an emulator with Id emu-7001. The milk tank CT has CT ID ct-2 and connects to the milk tank machine with machine ID “b8:22:eb:33:xx:xx”. The milk tank has a context associated with it. The context includes a description of the farm and its longitude and latitude information. Moreover, both the CTs support MQTT/TCP communication for machine data retrieval and sending. In the provided MQTT interfaces, the topics published by the CTs and subscribed to by the CTs are prefixed by the CT ID. They are in the format {CT-Id}/{MachineOutput}, {CT-ID}/{MachineInput} or {CT-ID}/{MachineSetting}. Moreover, the topics published by the emulator and subscribed by the emulator are both prefixed by the CT ID as well as the term “emu”. They are in the format {CT-ID}/emu/{MachineOutput}, {CT-ID}/emu/{MachineInput} or {CT-ID}/emu/{MachineSetting}. This is a convention used by the CT management framework for generating MQTT-based interfaces for CTs.

#### 5.2.2. Machine Simulators of the Milk Tank and Pickup Truck

To support this use case, we have developed custom simulators for the milk tank and the pickup truck. The CTs of the milk tank and the pickup truck each incorporate the instances of their respective simulators to support application testing. The hardware specifications of the developed simulators are as follows. The milk pickup truck simulator: QEMU-based Raspberry Pi hardware emulator proposed in [56] was used with configurations CPU: ARM1176, RAM: 256 MB and the operating system Raspbian Jezzie-lite [57]. Moreover, it was customized by adding a GPS sensor simulator that communicated with the Raspberry Pi hardware reflecting the 1-Wire communication protocol. The milk tank simulator: QEMU-based Raspberry Pi hardware emulator proposed in [56] was used with configurations CPU: ARM1176, RAM: 256 MB and the operating system Raspbian Jezzie-lite [57]. Moreover, it was customized by adding three simulators including a temperature sensor simulator, a quantity sensor simulator, and a tank control simulator that communicated with the Raspberry Pi hardware reflecting the 1-Wire communication protocol. The milk tank simulator also had the pickup event generator program that accepts pickup truck arrival events and generates the milk pickup events based on the data collected from the milk quantity and tank control simulators. Docker images were then built for each of the machine simulators by using Ubuntu 16.04.3 LTS as the base image. These simulator Docker images were utilized to conduct the experiments in Section 5.4. During the CT generation, the instances of the corresponding simulators were deployed along with the CTs.

Next, we describe the development of the milk pickup monitoring application using CTs.

### 5.3. CT-Based Milk Pickup Monitoring Application Development

Here we discuss the development activities involved in (a) a new CT-based milk pickup monitoring application, (b) the updating of the CT-based milk pickup application to a change of a pickup truck and (c) the porting of the CT-based milk pickup application to a different milk farm.

#### 5.3.1. Developing a New CT-Based Milk Pickup Monitoring Application

The application developer needs to use the web-based interface (described in Section 3.2) to find CTs and to query the machine descriptions of the milk tank and the pickup truck. It allows the application of filter criteria to find the CTs as well as allowing the querying of the CTs using SPARQL. As an example, to find the CT connecting to the pickup truck machine described in Figure 11, the application developer can provide the machine identification “b8:22:eb:77:xx:xx” as the filter criteria and then query the machine description as required to identify the required machine data.

**Figure 13:**
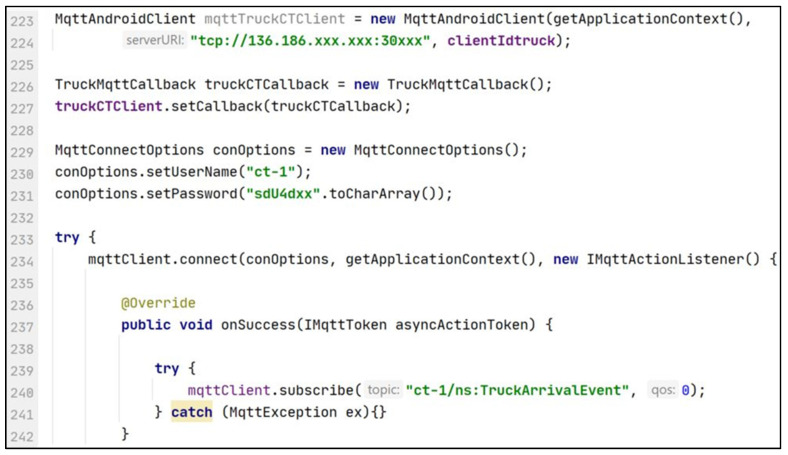
Integrating the pickup truck data with the milk pickup monitoring application via pickup truck CT.

After identifying the machine data needed by the application, the application developer can integrate the CTs and the related data with the application by connecting to an endpoint specified in the machine CT descriptions. As depicted in Figure 11 and Figure 12, the pickup truck CT, and the milk tank CT both provide MQTT-based endpoints to connect to the machines and their corresponding emulators via the CTs. The pickup monitoring application needs to receive truck arrival events from the pickup truck, and the milk temperature, milk quantity, and milk pickup events from the milk tank. Therefore, the application needs to subscribe to ct-1/ns:TruckArrivalEvent from ct-1 and ct-2/ns:MilkTemperature, ct-2/ns:MilkQuantity and ct-2/ns:PickupEvent topics from ct-2. The credentials for connecting to the CT endpoints are given in the CT descriptions. Figure 13 depicts the source code extracted from a milk pickup monitoring application developed using Android (Java). In the application, an existing MQTT library is used to integrate the truck CT with the application. As shown in line 240 of the code snippet, after successfully connecting to the truck CT, the application subscribes to the truck arrival event ct-1/ns:TruckArrivalEvent published by the truck CT. Similarly, the application can integrate the data from the milk tank CT.

**Figure 14:**
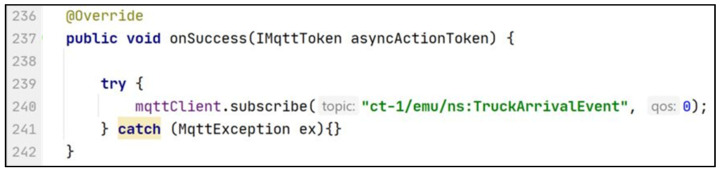
Using the pickup truck CT emulator for application testing.

Then the application functionality needs to be developed and the application needs to be tested. To test the application, the application developer can connect the application to the machine emulators/simulators via their CTs. As an example, to connect to the pickup truck’s emulator instead of the pickup truck the application only requires changing the subscribed topic from ct-1/ns:TruckArrivalEvent to ct-1/emu/ns:TruckArrivalEvent. Line 112 of Figure 13 needs to be changed as depicted in line 240 of Figure 14. Similarly, the application developer can switch between the milk tank and milk tank emulator via the CT.

#### 5.3.2. Updating an Existing CT-Based Milk Pickup Monitoring Application

Updating the application to a change of the pickup truck can be completed by updating the CT of the old pickup truck (i.e., ct-1) to connect with the new pickup truck. If the new pickup truck produces similar data as the old pickup truck, the application code does not need to be changed.

#### 5.3.3. Porting the CT-Based Milk Pickup Monitoring Application to a Different Milk Farm

If the pickup monitoring application is moved to a new farm, the application needs to be integrated with the pickup truck and the milk tank CTs in the new farm. The new farm has two CTs, ct-3 and ct-4, for the pickup truck and the milk tank, and they produce similar data as the previous machines. Now the application code needs to be changed to connect with the ct-3 and ct-4 instead of ct-1 and ct-2 via the respective interfaces provided by ct-3 and ct-4.

### 5.4. Experimentally Evaluating CT-Based Industry 4.0 Application Development

We used the milk pickup monitoring application for our evaluation. We first mapped the application to the cost model proposed in Section 4.2. Next, we provided an overview of the experimental methodology. Then we presented the costs of (a) developing a new Industry 4.0 application, (b) updating an existing Industry 4.0 application and (c) porting an existing Industry 4.0 application when using the CTs and (directly) using an existing IoT platform. Microsoft Azure is a leading IoT platform [58] and allows the development of Industrial solutions for Industry 4.0 [59]. Thus, we chose Azure IoT for our comparison. The environment ($p_{1}$) had a pickup truck $\left(m_{1}\right)$ and a milk tank ($m_{2}$). The pickup truck produces truck arrival events as machine output $\left(mo_{1}^{1}\right)$. It has a GPS sensor ($ms_{1}^{1,1})$ that produces machine output $(mo_{1}^{1}$). Milk tank ($m_{2}$) produces milk temperature $\left(mo_{1}^{2}\right)$, milk quantity $\left(mo_{2}^{2}\right)$, and pickup events $\left(mo_{3}^{2}\right)$ as machine outputs and accepts truck arrival events ($mi_{1}^{2}$) as machine inputs. It has a milk temperature sensor ($ms_{1}^{2,1})$ that produces machine output $\left(mo_{1}^{1}\right)$, milk quantity sensor ($ms_{2}^{2,2})$ that produces machine output $\left(mo_{2}^{1}\right)$, current sensor ($ms_{3}^{2})$ for detecting tank wash motor actuation, machine automation pickup event generator ($mc_{1}^{2})$ that produces machine output $\left(mo_{3}^{2}\right)$ and consumes truck arrival event machine input $\left(mi_{1}^{2}\right)$, and tank built-in control system $\left(mc_{2}^{2}\right)$ that actuates tank wash motor actuator ($ma_{1}^{2})$. These notations are summarized in Table 2.

#### 5.4.1. Experimental Setup and Methodology for Experiments

The CT-based application required the use of existing CTs of the machines that it required or the generation of the required CTs if they did not exist. We first used the CT management framework to generate any missing CT that was required by the application. The CT management framework components resided in a Kubernetes cluster master node with the configurations Ubuntu 20.04 LTS, RAM: 8 GB, 4VCPUs and Disk size 30GB. The Kubernetes cluster master node had two worker nodes registered with it each having configurations Ubuntu 20.04, RAM: 8 GB, 4VCPUs and Disk size 30 GB which were used by the CT management framework for deploying the CTs. Further details on CT generation and update processes are provided in [21]. The simulators described in Section 5.2.2 were also integrated by the CT management framework as a part of the CT generation for each of the CTs.

To compare the IoT platform-based and the CT-based Industry 4.0 implementations, we created an Azure IoT Hub instance and used that to integrate the machine simulators with the IoT platform. More specifically, in Industry 4.0 application, the pickup truck is represented as a simple machine that only generates GPS location data. To simulate this, we considered the Azure IoT -provided telemetry simulator [48] to generate device-simulated GPS data. On the other hand, in our application, the milk tank was a complex machine with machine automation. To simulate the milk tank machines we developed and integrated a milk tank simulator with the IoT platform. Milk tank simulators were deployed on a machine running Ubuntu 18.04 and with RAM size 8GB.

Next, we developed IoT platform-based and CT-based versions of the milk pickup monitoring application $a_{1}$ using the Python flask web application development framework. The user interfaces of these were developed using HTML. To minimize the impact on the final cost calculations from factors that did not specifically depend on these alternative implementations of $a_{1},$ we used the same user interfaces and the same data reading and writing mechanisms in the development of the IoT platform and the CT-based versions of $a_{1}$.

#### 5.4.2. Estimating the Costs of New Industry 4.0 Application Development

Developing $a_{1}$ requires the identification of the data required by $a_{1}$ from $m_{1}$ and $m_{2},$ including $mo_{1}^{1}$ of $m_{1}$ and $mo_{1}^{2},mo_{2}^{2},\;mo_{3}^{2}$ and $mi_{1}^{2}$ of $m_{2}$. It then requires the integration of these with $a_{1}$. Using $m_{1}$ and $m_{2}$ to develop $a_{1}$ requires coding the application functionality and testing it. To test $a_{1}$ it is necessary to simulate $m_{1}$ and $m_{2}$. $m_{1}$ is a simple machine with a single sensor $ms_{1}^{1,1}$. Therefore, it only requires the simulation of the sensor data and integrating it with $a_{1}$. The $m_{2}$ is a complex machine with machine automation $mc_{1}^{2}$. The $a_{1}$ interacts with $mc_{1}^{2}$ and it is required to simulate/emulate $m_{2}$ including $mc_{1}^{2}$ to support application testing. We calculated the application development cost by using Equation (1) as the basis. The assumptions made in the evaluation were:

- When developing the CT-based application we assumed that $m_{1}$ and $m_{2}$ were already integrated with their CTs. Therefore, the CT generation cost was not considered in the calculation of application development cost;
- In Azure IoT we assumed $m_{1}$ and $m_{2}$ were already integrated with the Azure IoT platform via the Azure IoT Hub [60]. Therefore, the cost of integration with the IoT platform was not considered in the calculation of the application development cost. Moreover, we assumed that $m_{1}$ and $m_{2}$ had sufficient and accurate machine descriptions associated with them that could be directly used by the application developer.

Next, we developed $a_{1}$ using the CTs of $m_{1}$ and $m_{2}$. Then we also developed $a_{1}$ using Azure IoT and integrated $m_{1}$ and $m_{2}$ and their data with the application via the IoT Hub endpoint. Table 3 summarizes the cost of application development using Azure IoT (first row) and CTs (second row). It shows the NoQs executed and the NoCs applied for identifying the data required from $m_{1}$ and $m_{2}$, SloC added or modified and NoCs applied for integrating $m_{1}$ and $m_{2}$ and the related data with the application and NoCs applied and SloC added or modified for developing and testing $a_{1}$. Equations (2), (6), and (10) proposed in Section 4.2 were used as the bases for calculating these values. In our calculations, we considered the values k_1_, k_2,_ and k_3_ introduced in Equations (5) and (9) to be the same with an average value of USD 18.00 [61].

**Figure 15:**
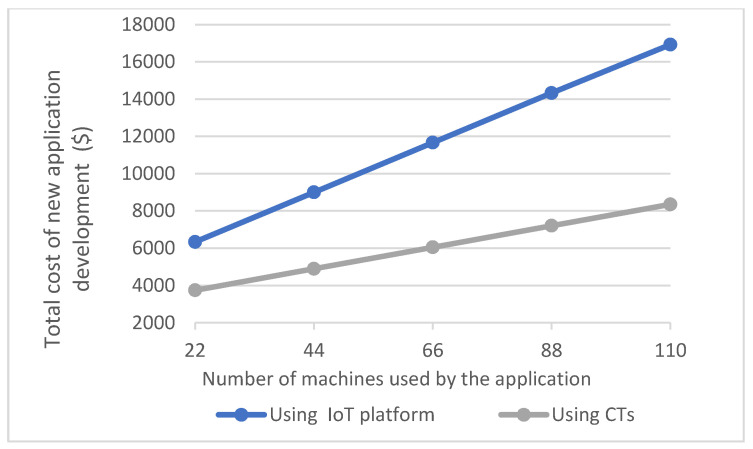
The new milk pickup monitoring application development cost and the number of machines used by the application.

Moreover, we next considered the following scenario to estimate the application development cost when the number of machines that needed to be utilized by $a_{1}$ changed. Consider a scenario where $a_{1}$ needs to monitor milk pickups in a set of milk farms in a region where each milk farm has a single milk tank with a ten thousand litres capacity and each pickup truck has a capacity of twenty thousand litres and makes five pickup trips per day. We estimated the application development cost when $a_{1}$ is developed for an environment having n milk tanks and n/10 pickup trucks for collecting milk, for n=20, n=40, n=60, n=80, n=100. Table 4 and Table 5 summarizes the cost estimates for Azure IoT and CTs, respectively. For each application that uses Azure IoT and CTs, the total cost was estimated as the summations of the cost of identifying the machine data, integrating the machines and the related data, and developing the application functionality and testing the application. The cost estimations for each of the application development activities were completed based on the results summarized in Table 3 and based on the Equations (2), (6) and (10) presented in Section 4.2. The line graph in Figure 15 further illustrates the related cost values.

#### 5.4.3. Estimating the Costs of Industry 4.0 Application Update

To evaluate the cost of updating an application, we considered a scenario where $m_{1}$ changed and $a_{1}$ needed to be integrated for a new pickup truck $\left(m_{3}\right)$. We considered that the models of both $m_{1}\;\mathrm{and}\;m_{3}$ were similar and provided similar machine outputs. The application update required the identification of data from $m_{3}$ ($mo_{1}^{3}$) and the integration of $m_{3}$ and $mo_{1}^{3}$ with the $a_{1}$. Equation (16) proposed in Section 4.2.4 was used to calculate the degree of adaptability of $a_{1}$. The assumptions made in the evaluation were:

- When updating $a_{1}$ to use $m_{3}$, we assumed that the CT of $m_{1}$ was updated to connect with $m_{3}$ without generating a new CT for $m_{3}$;
- When updating the application using Azure, we assumed that $m_{3}$ was already integrated with the same IoT Hub instance as $m_{1}$ and had a different device id.

**Figure 16:**
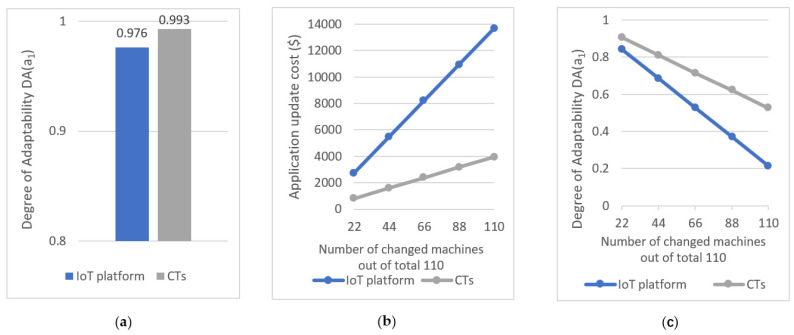
The milk pickup monitoring application: (**a**) Degree of adaptability of the application when using a single pickup truck and a milk tank; (**b**) Application update cost with the changed number of machines (**c**) Degree of the adaptability of the application with the changed number of machines.

Table 6 summarizes the cost of updating $a_{1}$ that used Azure IoT (first row) and CTs (second row), respectively. It shows the NoQs executed and the NoCs applied for identifying data from $m_{3}$, SloC added or modified, the NoCs applied for integrating $m_{3}$ and its data with $a_{1},$ and the NoCs applied and SloC added or modified for developing $a_{1}$ functionality and testing $a_{1}$. The final column summarizes the total cost of updating the application. Moreover, Figure 16a further illustrates the degree of adaptability of the two applications that were calculated using Equation (16).

Moreover, we next considered the following scenario to estimate the degree of adaptability of the pickup monitoring application with the number of changed/replaced machines when the application monitors an environment with hundred milk tanks and uses ten pickup trucks for collecting milk. We measured the degree of adaptability of the application when q machines out of the total 110 machines are changed/replaced, for q = 22, q = 44, q = 66, q = 88, q = 110. At each instance, we kept the number of changed milk tanks to the number of changed milk trucks ratio at 10:1. Table 7 (relating to Azure IoT) and Table 8 (relating to CTs) shows the costs involved in updating the pickup monitoring application when it utilizes 110 machines and some of them are changed or replaced. The cost estimations were completed based on the results summarized in Table 6 and the final column of Table 7 and Table 8 shows the degree of adaptability of the resulting application that was calculated using Equation (16). Figure 16b further illustrate the cost of updating the application and Figure 16c illustrates the degree of adaptability of the application with the number of changed machines.

#### 5.4.4. Estimating the Costs of Industry 4.0 Application Porting

To evaluate the portability of $a_{1}$ we considered a scenario where the $a_{1}$ was ported to a new environment ($p_{2}$) with a different milk tank ($m_{4}$) and a pickup truck ($m_{5}$). We considered the case where the models of $m_{4}$ and $m_{5}$ were similar to $m_{1}$ and $m_{2}$ in $p_{1}$, and produced similar machine outputs and accepted similar machine inputs. The degree of portability was calculated based on Equation (17) presented in Section 4.2.5. The assumptions made in the evaluation were:

- When porting $a_{1}$ that uses CTs to $p_{2}$ we assumed that CTs were available for $m_{4}$ and $m_{5}$;
- When porting $a_{1}$ using Azure IoT, we assumed $m_{4}$ and $m_{5}$ connected to a different IoT Hub and had different device ids, and they were already connected with the IoT Hub.

**Figure 17:**
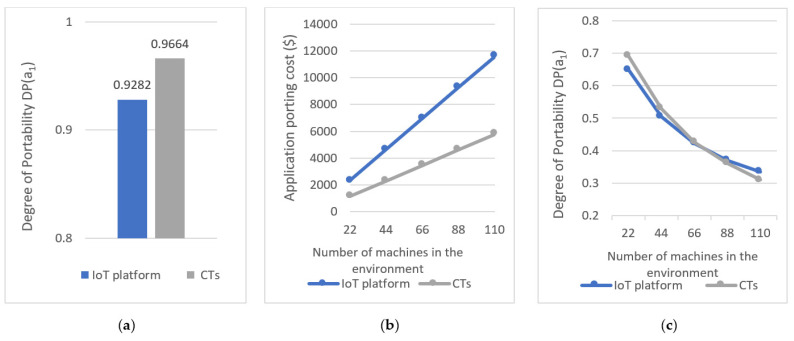
The milk pickup monitoring application, (**a**) Degree of portability of application for an environment with a single milk tank and a pickup truck; (**b**) Application porting cost with the number of machines used by the application, (**c**) Degree of portability of the application with the number of machines used by the application.

Table 9 summarizes the cost of porting $a_{1}$ that use Azure IoT and CTs. It shows the NoQs executed and the NoCs applied for identifying data from $m_{4}$ and $m_{5}$, SloC added or modified and NoCs applied for integrating $m_{4}$ and $m_{5}$ and their data with $a_{1}$ and NoCs applied and SloC added or modified for developing the functionality and testing of $a_{1}$. The final column summarizes the total cost of porting $a_{1}$. Moreover, Figure 17a further illustrates the degree of portability of the two applications.

Moreover, we next considered the following scenario to estimate the degree of portability of the pickup monitoring application $a_{1}$ with the number of machines (i.e., milk tanks and milk trucks) utilized by the application. The degree of portability of the application was measured when the application needs to be ported from $p_{1}$ to $p_{2}$ where $p_{1}$ and $p_{2}$ each is having n milk tanks and n/10 pickup trucks, for n=20, n=40, n=60, n=80, n=100. The cost estimates were completed based on the results summarized in Table 9 and Table 10 (relating to Azure IoT) and Table 11 (relating to CTs) summarizes the estimated costs involved in porting $a_{1}$ when the number of machines in the environment that is used by the application changes from 22 to 110. The final column of both tables shows the resulting degree of portability of the applications that were calculated based on Equation (17). Figure 17b further illustrates the cost of porting the CT-based application and the application developed using Azure IoT. Figure 17c further illustrates the change in the degree of portability.

### 5.5. Discussion

Figure 15 illustrates the application development costs using Azure IoT and CTs with the number of machines used by the application. The application development cost shows a gradual incremental increase in the CT-based approach with the increase in the number of machines used. However, the cost of (directly) using Azure IoT is high when compared to using CTs. This is because Azure requires additional effort to integrate the milk tank emulator with the IoT platform to support application testing. This can be also seen in Table 3, as the effort of developing and testing the application that uses Azure IoT is relatively high (SLoC 159) when compared to the application that uses CTs (SLoC 112). Moreover, it can be observed that there is a sharp increase in the cost of development using Azure IoT compared to using CTs. Moreover, in these instances, the CT-based application development cost is approximately 60% or less than the IoT platform-based Industry 4.0 application development cost. Hence, we can conclude that using CTs to develop the pickup monitoring application is more cost-efficient.

The results presented in Figure 16a show the degree of adaptability of the pickup monitoring application developed using the Azure IoT and CTs for an environment with a single pickup truck and a milk tank. The application that utilizes CTs has a higher degree of adaptability when compared to the application that uses the Azure IoT. This is because CT offers increased adaptability when the corresponding physical machine is changed. This reduces the integration cost of the new machine, and it is primarily achieved by updating the CT. However, the application that uses the IoT platform requires the integration of the new machine and its data with the application. Figure 16b shows the application update cost using Azure IoT and CTs with a changed number of machines. As shown in the figure, updating an application is less costly than developing a new application in both Azure IoT and when using CTs. However, the application update cost is high when using the Azure IoT platform compared to using CTs. The results presented in Figure 16c compares the degree of adaptability of the pickup monitoring application that utilizes 100 milk tanks and 10 milk trucks when some of the machines used by the application are changed. In the figure, it can be seen that the applications that use CTs are more adaptable to change of machines when compared to the application that uses the IoT platform. In summary, the application that uses CTs offers a higher degree of adaptability, and the update cost of CT-based applications is less.

The results presented in Figure 17a show the degree of portability of the pickup monitoring application developed using the Azure IoT and CTs for an environment with a single milk tank and a pickup truck. The application that utilizes the CTs has a higher degree of portability when compared to the application that uses the IoT platform. The results presented in Figure 17b compares the cost of porting the application that uses Azure IoT and CTs with the number of machines used by the application. As shown in the figure, the cost is less with CTs. The results presented in Figure 17c compare the degree of portability of the pickup monitoring application when the number of machines used by the application is increased starting from 20 milk tanks and 2 pickup trucks up to 100 milk tanks and 10 pickup trucks. The application that uses CTs and Azure seems to have a similar degree of portability. However, the porting cost and redevelopment cost of an application that uses CTs is less when compared to the cost of porting an application that uses Azure IoT. Thus, the porting cost of CT-based Industry 4.0 application is less compared to the IoT platform-based application. In summary, it can be concluded that the CT-based Industry 4.0 application development, update and porting is more cost-efficient when compared to using an IoT platform.

## 6. Conclusions and Future Research

In this paper, we proposed CTs for CT-based Industry 4.0 application development and introduced a novel cost model for estimating the cost of developing Industry 4.0 applications. We also presented an experimental evaluation of CT-based Industry 4.0 application using a case study from the dairy industry that illustrated the benefits of CT-based Industry 4.0 application development in comparison with traditional IoT platform-based application development. In this evaluation, we considered all aspects of the application development lifecycle including the development of a new Industry 4.0 application, the updating of an existing Industry 4.0 application to a change of a machine and porting an existing Industry 4.0 application to a different set of machines (e.g., deployment to a new plant). We used the proposed cost model to compare the application development costs for each application development scenario when using the CTs and (directly) using an IoT platform. This evaluation showed that CT-based application development is less costly than the IoT platform-based alternative when the same CTs are used to develop multiple Industry 4.0 applications, which is normally the case in all industry settings that require efficiency, preventive maintenance, and product consistency management and improvement.

In our future work, we aim to extend our experimental analysis to include a statistics-based approach to validate the significant improvement in cost of developing the Industry 4.0 application and the degree of adaptability of the Industry 4.0 application using the proposed CTs-based approach compared to IoT platform-based approaches. We also aim to extend the CTs, the CT management framework and the cost model to support complex machines which incorporate automation that determines the machine actuation and the data that the machine will generate based on the automation. In particular, we aim to explore CT model extensions that are symbiotic to the automation in complex machines.

## Figures and Tables

**Table 1:** Notations and their description.

Notation	Description
A	Set of applications ($a_{l}\in A$)
M	Set of machines in a plant ($m_{j}\in M$)
I	Set of inputs
O	Set of outputs
$M^{l}$	Set of machines that are utilized by $a_{l}$ ($M^{l}\subset M$)
$I^{l,j}$	Set of inputs provided by $a_{l}$, to $m_{j}$
$O^{l,j}$	Set of outputs retrieved by $a_{l}$, from $m_{j}$
$MS^{j}$	Set of sensors in $m_{j}$
$MA^{j}$	Set of actuators in $m_{j}$
$MC^{j}$	Set of machine automation/machine controls in $m_{j}$
$MI^{j}$	Set of machine inputs consumed by $m_{j}$ ($mi_{p}^{j}\in MI^{j}$)
$MO^{j}$	Set of machine outputs produced by $m_{j}$ ($mo_{q}^{j}\in MO^{j}$)
$MST^{j}$	Set of machine settings consumed by $m_{j}$ ($mst_{r}^{j}\in MST^{j}$)
T	Set of data translators
$T^{l,j}$	Set of data translators used by $a_{l}$ for translating machine outputs produced by $m_{j}$
$Cost_{Total}\left(a_{l}\right)$	The total cost of developing $a_{l}$ by using $M^{l}$
$Cost_{identify}\left(M^{l},a_{l}\right)$	Cost of identifying the data needed by $a_{l}$ from $M^{l}$
$Cost_{int}\left(M^{l},a_{l}\right)$	Cost of integrating $M^{l}$ and their data with $a_{l}$
$Cost_{dt}\left(M^{l},a_{l}\right)$	Cost of developing and testing $a_{l}$ using $M^{l}$

**Table 2:** Summary of notations.

Notation	Description
$p_{1}$	Environment being monitored
$a_{1}$	Pickup monitoring application
$m_{1}$	Pickup truck
$mo_{1}^{1}$	Machine outputs of $m_{1}$
$m_{2}$	Milk tank
$mo_{1}^{2},mo_{2}^{2},\;mo_{3}^{2}$	Machine outputs of $m_{2}$
$mi_{1}^{2}$	Machine input of $m_{2}$
$mc_{1}^{2}$	Pickup event generator machine automation of $m_{2}$

**Table 3:** Cost of developing the pickup monitoring applications using the IoT platform and CTs.

Industry 4.0 Application	Identify (NoQ)	Identify (NoC)	Integrate (NoC)	Integrate (SloC)	Dev and Test (NoC)	Dev and Test (SloC)	Total Cost ($)
IoT platform	0	2	4	42	2	159	3762.00
CTs	0	2	3	32	0	112	2682.00

**Table 4:** Cost of developing the pickup monitoring application (directly) using IoT platform and the number of machines used by the application.

Number of Milk Tanks	Number of Milk Trucks	Cost of Identifying	Cost of Integration	Cost of Dev and Test	Total Cost ($)
20	2	396.00	2286.00	4050.00	6732.00
40	4	792.00	3402.00	5202.00	9396.00
60	6	1188.00	4518.00	6354.00	12,060.00
80	8	1584.00	5634.00	7506.00	14,724.00
100	10	1980.00	6750.00	8658.00	17,388.00

**Table 5:** Cost of developing the CT-based pickup monitoring application and the number of machines used by the application.

Number of Milk Tanks	Number of Milk Trucks	Cost of Identifying	Cost of Integration	Cost of Dev and Test	Total Cost ($)
20	2	396.00	1332.00	2016.00	3744.00
40	4	792.00	2088.00	2016.00	4896.00
60	6	1188.00	2844.00	2016.00	6048.00
80	8	1584.00	3600.00	2016.00	7206.00
100	10	1980.00	4356.00	2016.00	8352.00

**Table 6:** Cost of updating the pickup monitoring application using the IoT platform and CTs.

Industry 4.0 Application	Identify (NoQ)	Identify (NoC)	Integration (NoC)	Integration (SloC)	Dev and Test (NoC)	Dev and Test (SloC)	Total Cost ($)
IoT platform	0	1	0	2	3	0	90.00
CTs	0	1	1	0	0	0	36.00

**Table 7:** Degree of adaptability of the pickup monitoring application developed using the IoT platform with the changed number of machines.

Changed Machines/Total No. of Machines	Cost of Updating	Cost of Redev.	Degree of Adaptability
22/110	2736.00	17,388.00	0.8426
44/110	5472.00	17,388.00	0.6853
66/110	8208.00	17,388.00	0.5279
88/110	10,944.00	17,388.00	0.3706
110/110	13,680.00	17,388.00	0.2133

**Table 8:** Degree of adaptability of the CT-based pickup monitoring application with the changed number of machines.

Changed Machines/Total No. of Machines	Cost of Updating	Cost of Redev.	Degree of Adaptability
22/110	792.00	8352.00	0.9052
44/110	1584.00	8352.00	0.8103
66/110	2376.00	8352.00	0.7156
88/110	3168.00	8352.00	0.6207
110/110	3960.00	8352.00	0.5259

**Table 9:** Cost of porting the pickup monitoring applications using the IoT platform and CTs.

Industry 4.0 Application	Identify (NoQ)	Identify (NoC)	Integration (NoC)	Integration (SLoC)	Dev and Test (NoC)	Dev and Test (SLoC)	Total Cost ($)
Azure IoT	0	2	4	3	2	4	270.00
CTs	0	2	3	0	0	0	90.00

**Table 10:** The degree of portability of the pickup monitoring application developed by (directly) using the IoT platform and the number of machines in the environment.

No. of Machines	Cost of Porting	Cost of Redev.	Degree of Portability
22	2358.00	6732.00	0.6497
44	4662.00	9396.00	0.5039
66	6966.00	12,060.00	0.4224
88	9270.00	14,724.00	0.3704
110	11,574.00	17,388.00	0.3344

**Table 11:** The degree of portability of the CT-based pickup monitoring application and the number of machines in the environment.

No. of Machines	Cost of Porting	Cost of Redev.	Degree of Portability
22	1152.00	3744.00	0.6923
44	2304.00	4896.00	0.5294
66	3456.00	6048.00	0.4256
88	4608.00	7200.00	0.3600
110	5760.00	8352.00	0.3103

## Data Availability

Not applicable.
